# *N*-Acetylcysteine Reverses the Mitochondrial Dysfunction Induced by Very Long-Chain Fatty Acids in Murine Oligodendrocyte Model of Adrenoleukodystrophy

**DOI:** 10.3390/biomedicines9121826

**Published:** 2021-12-03

**Authors:** Jie Zhou, Marcia R. Terluk, Paul J. Orchard, James C. Cloyd, Reena V. Kartha

**Affiliations:** 1 Center for Orphan Drug Research, Department of Experimental and Clinical Pharmacology, College of Pharmacy, University of Minnesota, 2001 6th Street SE, Minneapolis, MN 55455, USA; zhoux383@umn.edu (J.Z.); mrterluk@umn.edu (M.R.T.); cloyd001@umn.edu (J.C.C.); 2 Division of Pediatric Blood and Marrow Transplantation, Department of Pediatrics, Medical School, University of Minnesota, 425 East River Parkway, Minneapolis, MN 55455, USA; orcha001@umn.edu

**Keywords:** adrenoleukodystrophy (ALD), very long-chain fatty acids (VLCFA), antioxidant, *N*-acetylcysteine (NAC), glutathione (GSH), mitochondrial GSH (mtGSH), mitochondrial dysfunction, oligodendrocytes

## Abstract

The accumulation of saturated very long-chain fatty acids (VLCFA, ≥C22:0) due to peroxisomal impairment leads to oxidative stress and neurodegeneration in X-linked adrenoleukodystrophy (ALD). Among the neural supporting cells, myelin-producing oligodendrocytes are the most sensitive to the detrimental effect of VLCFA. Here, we characterized the mitochondrial dysfunction and cell death induced by VLFCA, and examined whether *N*-acetylcysteine (NAC), an antioxidant, prevents the cytotoxicity. We exposed murine oligodendrocytes (158 N) to hexacosanoic acid (C26:0, 1–100 µM) for 24 h and measured reactive oxygen species (ROS) and cell death. Low concentrations of C26:0 (≤25 µM) induced a mild effect on cell survival with no alterations in ROS or total glutathione (GSH) concentrations. However, analysis of the mitochondrial status of cells treated with C26:0 (25 µM) revealed depletion in mitochondrial GSH (mtGSH) and a decrease in the inner membrane potential. These results indicate that VLCFA disturbs the mitochondrial membrane potential causing ROS accumulation, oxidative stress, and cell death. We further tested whether NAC (500 µM) can prevent the mitochondria-specific effects of VLCFA in C26:0-treated oligodendrocytes. Our results demonstrate that NAC improves mtGSH levels and mitochondrial function in oligodendrocytes, indicating that it has potential use in the treatment of ALD and related disorders.

## 1. Introduction

X-linked adrenoleukodystrophy (ALD), a progressive, debilitating, and often fatal disorder which occurs due to genetic mutations in ABCD1, a peroxisomal membrane transporter of very long-chain fatty acids (VLCFA) composed of 22 or more carbon atoms [1]. As a result, regular transport of VLCFA into peroxisomes and its subsequent degradation is impaired, causing its intracellular accumulation, leading to lipotoxicity. Increased levels of VLCFA such as hexacosanoic acid (C26:0) have been observed in the brain and adrenal tissues of ALD patients [2]. The cellular mechanisms by which VLCFA accumulation contributes to ALD onset and progression are still unclear. However, it is recognized that there is an urgency to identify novel molecules that can improve the management of patients with ALD. Emerging evidence indicates a close relationship between VLCFA accumulation and mitochondrial dysfunction, with the rate of VLCFA β-oxidation being dependent on mitochondrial status [3]. More recently, decreased mitochondrial function parameters have been observed in in vitro and in vivo ALD models [4,5,6,7]. Mitochondria have antioxidant defenses such as glutathione (GSH) and detoxifying enzymes to attenuate oxidative stress [8]. Further, mitochondria play a central role in initiating and regulating programmed cell death or apoptosis [9]. Thus, even if the primary pathology of ALD is unrelated to mitochondria, dysfunction of mitochondria is a significant secondary factor in determining clinical outcomes [10].

Antioxidants have been widely used in the restoration of mitochondrial function [11,12,13,14]. Specifically, *N*-acetylcysteine (NAC), a prodrug for cysteine, is a well-known antioxidant and a precursor for the crucial intracellular antioxidant GSH [15] that protects against cellular damage caused by free radicals [16]. NAC has been shown to elevate brain GSH levels, indicating its therapeutic potential in psychiatric and neurological disorders [17,18]. NAC plays a protective role by targeting mitochondrial impairment in age-associated neurodegenerative disorders such as Alzheimer’s disease [19], Parkinson’s disease [20], and mental health disorders [17]. Additionally, NAC was shown to effectively suppress lipid peroxidation-induced mitochondrial injury in primary neuronal cells [21].

Since oligodendrocyte death is considered an early event in central nervous system (CNS) demyelination and neurodegeneration [22], this study was designed to characterize the toxic effects of C26:0 on these cells using an established murine oligodendrocytes cell line (158 N) [23]. Further, we examined the potential therapeutic effect of NAC on reversing the toxicity of VLCFA on mitochondria function.

## 2. Materials and Methods

### 2.1. Materials

Dulbecco’s Modified Eagle Medium (DMEM) high glucose media, fetal bovine serum (FBS), antibiotic-antimycotic (AA) solution, phosphate-buffered saline (PBS), Trypsin-EDTA, fluorescent CM-H_2_DCFDA, and MitoSOX^TM^ probes were obtained from Life Technologies (Carlsbad, CA, USA). The 7-AAD fluorescent probe was purchased from BD Biosciences (San Jose, CA, USA). Hexacosanoic acid (C26:0), acrolein, *N*-acetylcysteine (NAC), sucrose, mannitol, and ethylene glycol-bis (2-aminoethylether)-*N*,*N*,*N*′,*N*′-tetra acetic acid (EGTA) were purchased from Millipore Sigma (St. Louis, MO, USA). HEPES 1 M solution was from Mediatech (Manassas, VA, USA).

### 2.2. Cell Culture and Treatments

The immortalized murine oligodendrocyte cell line, 158 N (normal) was a generous gift from Dr. Ghandour [23]. 158 N cells were cultured in 75 cm^2^ flasks in DMEM high glucose media supplemented with 5% FBS and 1% AA at 37 °C and 5% CO_2_. For experimental setup, cells were seeded into 75 cm^2^ culture flasks, 6-well, 24-well, or 96-well plates, and kept overnight to achieve ~80% confluence. Cells were challenged for 24 h with increasing concentration of C26:0 from 1 to 100 μM. In another set of experiments, NAC at concentrations ranging from 50 to 500 µM was co-incubated with C26:0 for 24 h. Cells treated with 1–2% ethanol served as control or vehicle groups. C26:0 was dissolved in 100% ethanol (heated to 37 °C) to make a 2.5 mM stock solution. The pH of the NAC stock solution (10 mM in PBS solution) was adjusted to 7.4 and filtered before making further dilutions.

### 2.3. Cell Survival Assay

Cell survival colorimetric assay was performed in a 96-well plate using Cell Counting Kit-8 from Dojindo (Kumamoto, Japan) according to the manufacturer’s instructions. The absorbance was read at 450 nm with a Synergy 2 microplate reader and Gen 5 software (Biotek, Winooski, VT, USA). The results were calculated as a percentage of the control cells, which was considered 100% viability.

### 2.4. Measurement of Intracellular ROS Production

Evaluation of ROS was measured by fluorescence-activated cell sorting (FACS) using fluorescent CM-H_2_DCFDA probes as previously described [24]. Briefly, after 24 h of culture in varying conditions, cells were harvested, washed, and stained with 1 µM CM-H_2_DCFDA for 5 min. The samples were subsequently washed and resuspended in 250 µL PBS containing 5 µL 7-AAD fluorescent probes for analysis. During the acquisition of FACS data, the live cells (negative for 7-AAD) were gated and evaluated for ROS with CM-H_2_DCFDA. Further, the fluorescence intensity of CM-H_2_DCFDA was also gated uniformly to designate positive events of CM-H_2_DCFDA stained cells. The fluorescence of CM-H_2_DCFDA was measured at excitation at 485 nm and emission at 530 nm. The percentage of positive stained CM-H_2_DCFDA was used as the indicator for ROS levels in different treatment groups.

### 2.5. Determination of Total GSH Content

Cells seeded on 24-well plates were subjected to the experimental treatments as previously described. At the end of 24 h incubation, cells were washed twice with PBS and the cell lysates were collected by using lysis buffer (20 mM HEPES, 1 mM EGTA, 210 mM mannitol, and 70 mM sucrose, pH 7.2). Total GSH was quantified using Glutathione Assay Kit (Cayman Chemical, Ann Arbor, MI, USA) at 405–414 nm wavelength following the manufacturer’s protocol. Total GSH levels were normalized by the protein amount (µg/mg of total protein) and were quantified by Bradford protein assay. Further, relative total GSH was calculated by dividing the GSH concentration of the treatment groups by the control.

### 2.6. Mitochondrial Inner Membrane Potential

Mitochondrial inner membrane potential was measured using JC-1 Mitochondrial Membrane Potential Assay Kit (Cayman Chemical, Ann Arbor, MI, USA) according to the manufacturer’s protocol. We quantitated the staining using a fluorescence microplate reader (SpectraMax, Molecular Devices, San Jose, CA, USA) and visualized the stained cells using a Zeiss Axiovert 200 M fluorescence microscope (Carl Zeiss AG, Oberkochen, Germany). The excitation/emission wavelength was set up as follows: J-aggregates (red): 560/595 nm; JC-1 monomers (green): 485/535 nm.

After 24 h of culture in varying conditions, cells seeded on 6-well plates were stained with JC-1 staining solution prepared per protocol for 30 min under dark conditions. The cells were subsequently washed twice using a culture medium and continually cultured in a fresh culture medium for imaging. Digital images were taken by an inverted fluorescence microscope (Zeiss Axiovert 200 M) fitted with a matched Axiocam 4-megapixel camera. Images were captured using a 20× objective lens (N.A. 0.3) to visualize the JC-1 monomers inside the mitochondria. In addition, a fluorescence microplate reader was used to quantify the J-aggregates as well as JC-1 monomers in following cell treatments in 96-well culture plates. The ratio of fluorescent intensity of J-aggregates to fluorescent intensity of monomers was used as an indicator of cell health and calculated for each well.

### 2.7. Mitochondrial Superoxide Levels

MitoSOX red reagent (Life Technologies, Carlsbad, CA, USA) was used to quantify superoxide anion produced in the mitochondria. MitoSOX permeates live cells, targets mitochondria, and exhibits red fluorescence once oxidized by superoxide anion. MitoSOX stained cells were measured by fluorescence microscopy per the manufacture’s protocol. After 24 h of culturing, cells seeded on 6-well plates were stained with 5 µM MitoSOX staining solution prepared per protocol for 10 min under dark conditions. The cells were subsequently washed gently three times using the culture medium and continually cultured in a fresh culture medium for imaging. Following the various treatments, digital phase-contrast and MitoSOX fluorescence images were observed after 24 h under an inverted fluorescence microscope, camera and a 20× objective lens, as described above. Representative digital images were obtained from three independent experiments. The excitation/emission spectra for MitoSOX were set up at 510/580 nm, following the manufacturer’s recommendations.

### 2.8. Mitochondrial Glutathione Levels

Mitochondria were isolated using Mitochondria Isolation Kit for Cultured Cells (Thermo Scientific, Waltham, IL, USA) following experimental treatments. Isolated mitochondria were further lysed with buffer (20 mM HEPES, 1 mM EGTA, 210 mM mannitol, 70 mM sucrose, and pH 7.2) and mitochondrial GSH (mtGSH) levels measured using Glutathione Assay Kit from Cayman Chemical (Ann Arbor, MI, USA) as previously described. Total protein levels were measured as before using Quick Start Bradford Protein Assay from Bio-Rad (Hercules, CA, USA) and mtGSH levels were expressed as µg/mg total protein. mtGSH levels in cells treated with culture medium served as control and were used to normalize across different groups.

### 2.9. ATP Content Analysis

ATPlite™ Luminescence Assay System from PerkinElmer (Waltham, MA, USA) was used to detect ATP content as per the manufacturer’s protocol on 24-well cell culture plates.

### 2.10. Statistical Analysis

For all cellular assays, results were expressed as means ± standard error. Data were analyzed using ANOVA with a Bonferroni’s correction for multiple comparisons and Dunnett’s test when multiple comparisons were made against the control. A *p*-value < 0.05 was considered significant. Analyses are based on data from three independent experiments performed in triplicates using different cell passages on different days. All statistical analysis was performed using software in GraphPad Prism 8 (GraphPad Software, Inc., La Jolla, CA, USA).

## 3. Results

### 3.1. VLCFA Affects Cell Survival and Mitochondrial Status in Oligodendrocytes

**Figure 1:**
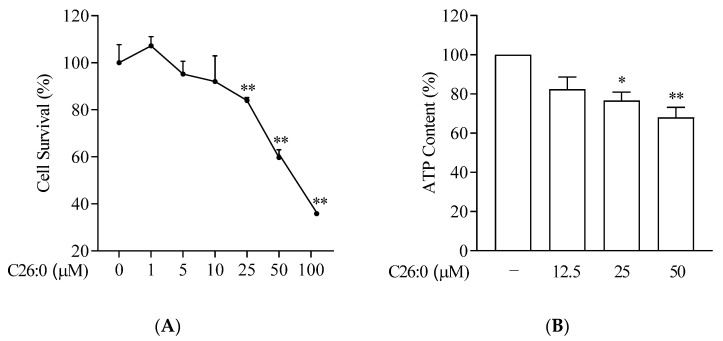
C26:0 reduced cell survival and ATP production in a concentration-dependent manner. (**A**) Percentage of viable 158 N cells after incubation with C26:0 (0–100 µM) for 24 h are shown (**A**, *n* = 6). At concentrations of C26:0 equal to and higher than 25 µM, significant cell death was observed, ** *p* < 0.01, ANOVA followed by Dunnett’s test. The 1% ethanol group is shown as vehicle control. (**B**) Percentage of ATP content after incubation with C26:0 (0–50 µM) for 24 h (**B**, *n* = 6). At concentrations of C26:0 equal to and higher than 25 µM, a significant decrease in ATP production compared to ethanol control was observed, * *p* < 0.05, ** *p* < 0.01, ANOVA followed by Dunnett’s test. The equations of the standard curve were used to calculate the concentration of ATP per well and the results are expressed as a percentage of control.

**Figure 2:**
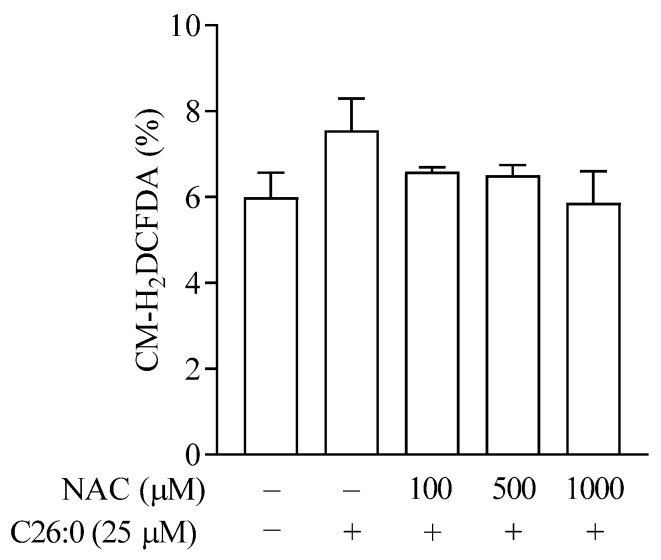
ROS levels in 158 N cells after incubation with C26:0 and NAC for 24 h were measured (*n* = 3) by H_2_DCFDA. No statistically significant change was observed between 1% ethanol control and 25 µM C26:0 treatment (*t*-test, *p* =0.17). Co-incubation of NAC (100–1000 µM) and 25 µM C26:0 treatment did not change cellular ROS levels.

To characterize the effect of VLCFA in oligodendrocytes, we first examined the effect of increasing concentrations of hexacosanoic acid (C26:0, 1 to 100 µM) on cell viability and intracellular ATP production. The survival of 158 N cells was not affected significantly following incubation with C26:0 at 1 to 10 µM (Figure 1A). However, the cell survival rates were significantly lower at 25 µM (84 ± 3%), 50 µM (60 ± 8%) and 100 µM (18 ± 1%) concentrations of C26:0, compared to vehicle control (*p* < 0.01). We also observed a concentration-dependent decrease in ATP production following treatment with C26:0 (Figure 1B), showing the toxicity of VLCFA on the cells. Since mitochondria are the major source of cellular ATP, we explored overall mitochondrial function further. It is noteworthy that at 25 µM concentration, C26:0 only marginally affected cell survival (~15% reduction), whereas there was a significant reduction in ATP production as compared to vehicle control (*p* < 0.05). Based on both these observations, we concluded that the lower concentration limit for C26:0 toxicity in 158 N cells was around 25 µM. Next, we evaluated whether this threshold VLCFA concentration can induce cellular oxidative stress in oligodendrocytes and if antioxidant NAC can mitigate this process. We observed an increasing trend in ROS formation compared to vehicle control (1% ethanol), which was normalized by increasing concentrations of NAC (Figure 2).

### 3.2. NAC Prevents Mitochondrial GSH (mtGSH) Depletion Induced by VLCFA

**Figure 3:**
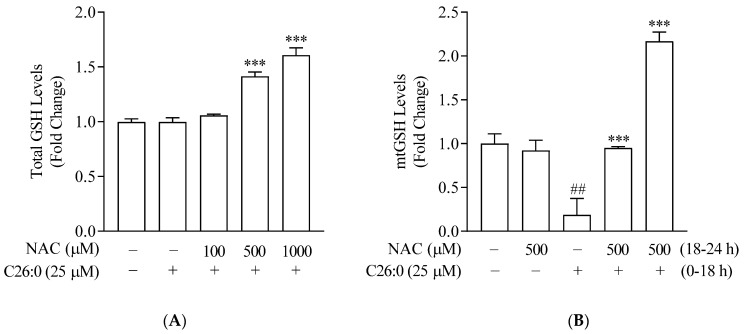
Total intracellular GSH (**A**) and mtGSH levels (**B**) in 158 N cells were measured after incubation with C26:0 and NAC for 24 h (*n* = 3). A) No statistically significant change in total GSH was observed between control and C26:0 (25 µM) (*t*-test, *p* = 0.99). Co-incubation of NAC (100–1000 µM) and 25 µM C26:0 treatment increased total GSH in a concentration-dependent manner (**A**; *** *p* < 0.001, ANOVA followed by Dunnett’s test). (**B**) Significant decrease in mtGSH was observed between control and C26:0 (25 µM) (*t*-test, ## *p* <0.01). Additional co-incubation of NAC (500 µM) restored mtGSH depleted by C26:0 (*** *p* < 0.001 indicates a significant increase in mtGSH with co-incubation of NAC compared to C26:0 (25 µM) treatment only; ANOVA followed by Bonferroni’s test). Moreover, mtGSH was increased to 2.2 ± 0.1-fold of control when cells were incubated with C26:0 (25 µM) for 18 h followed by NAC (500 µM) for 6 h. The 1% ethanol-treated cells served as control.

We characterized the effect of VLCFA on endogenous antioxidant GSH and the benefit of NAC, by analyzing both intracellular total GSH and mtGSH levels. The exposure of 158 N cells to 25 µM C26:0 for 24 h did not cause any depletion in total GSH (Figure 3A, *p* = 0.99) as compared to vehicle control. This further confirmed 25 µM as the threshold concentration of C26:0, which was selected for further studies to understand the mitochondria-targeted effect of C26:0. The addition of NAC in presence of threshold C26:0 levels resulted in a significant increase of total cellular GSH in a concentration-dependent manner.

We then conducted a series of experiments to evaluate changes in mitochondrial status, such as mtGSH, superoxide anions, and inner membrane potential (∆ψ_m_) after incubating with C26:0. Either mitochondria were isolated or a mitochondrial-specific probe was used in intact cells. Incubation with 25 µM C26:0 resulted in a significant depletion of mtGSH (0.2 ± 0.2-fold of vehicle control, *p* < 0.01, Figure 3B). This is in sharp contrast to our previous observation on intracellular total GSH (0.99 ± 0.04-fold of vehicle control, *p* = 0.99, Figure 3A). This suggests that the nominal accumulation of VLCFA has an early effect on the mitochondrial antioxidant defense system before exerting the toxic effects elsewhere. We then examined whether NAC could reverse this mitochondrial-specific toxic effect of VLCFA. Our results indicate that NAC at 500 µM added along with C26:0 can replenish the mtGSH levels to control levels (0.95 ± 0.02 fold of vehicle control) with significant increases in intracellular total GSH (1.4 ± 0.04 fold of vehicle control, *p* < 0.001, Figure 3A,B). To examine whether NAC can protect cells previously exposed to VLCFA, we pretreated cells with C26:0 (25 µM, 18 h) followed by NAC (500 µM, 6 h). We observed a 2.2 ± 0.1-fold induction in mtGSH levels compared to control (*p* < 0.001) (Figure 3B). Together we have shown that NAC provides cellular protection against the C26:0 accumulation primarily by replenishing mtGSH.

### 3.3. NAC Reverses the Increase in Mitochondrial Superoxide Caused by VLCFA 

**Figure 4:**
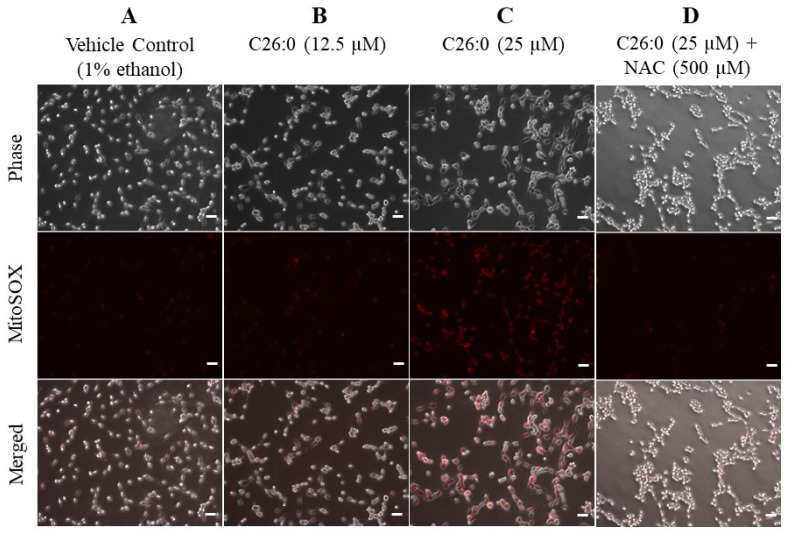
C26:0 increased mitochondrial superoxide level which was mitigated by NAC. (**A**–**D**) Representative images of 158 N cells show an increase in MitoSOX fluorescence following treatment with C26:0 (0, 12.5 and 25 µM) for 24 h. Additional co-incubation of NAC (500 µM) notably decreased MitoSOX fluorescence, indicating reduced levels of mitochondrial superoxide formation in presence of NAC. Scale bar (-) in white represents 50 µm.

Superoxide anions are produced mainly in the mitochondria, generating other ROS such as hydroxyl radical and hydrogen peroxide [25]. We next used MitoSOX red reagent to detect the formation of superoxide anion following treatment with C26:0. We observed superoxide anions to accumulate with increasing concentrations of C26:0 (Figure 4A–C). This indicates that VLCFA increased superoxide anion levels within mitochondria due to exacerbated superoxide anion production and/or an impaired antioxidant defense system. We then evaluated whether the increase in mitochondrial superoxide is mitigated by NAC. The addition of 500 µM NAC notably decreased MitoSOX fluorescence (Figure 4D). Our results indicate that treatment with NAC can effectively moderate ROS accumulation within mitochondria.

### 3.4. NAC Alleviates VLCFA-Induced Impairment in Mitochondrial Inner Membrane Potential

**Figure 5:**
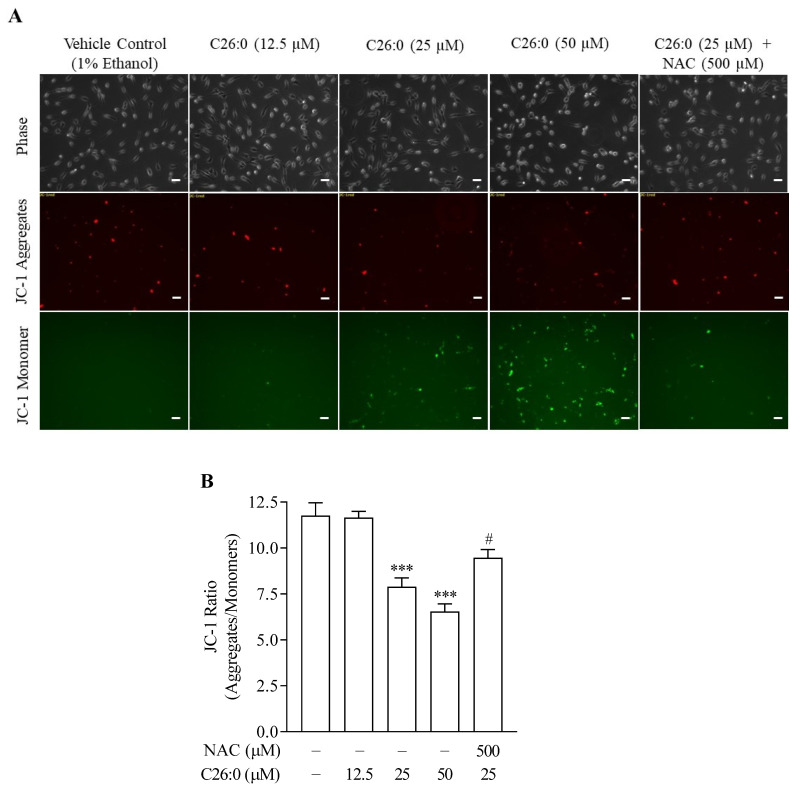
C26:0 decreased mitochondrial inner membrane potential (∆ψ_m_), which was alleviated by NAC. (**A**) Representative images of 158 N cells show an increase in JC-1 monomers (green fluorescence in dead cells) and corresponding decrease in JC-1 aggregates (red fluorescence in healthy cells) following C26:0 (0–50 µM) incubation for 24 h. Additional co-incubation of NAC increased red fluorescence and decreased green fluorescence, indicating restoration of ∆ψ_m_. Scale bar (−) in white represents 50 µm. (**B**) Bar plot indicates quantification of JC-1 fluorescence in 96-well plates. The ∆ψ_m_ decreased significantly following incubation with C26:0 (25 µM and 50 µM) compared to control (*** *p* < 0.001, ANOVA followed by Dunnett’s test). This mitochondrial toxicity was reversed by NAC (# *p* < 0.05, ANOVA followed by Bonferroni’s test).

To further characterize the detrimental effect of VLCFA on mitochondrial function, we also measured mitochondrial inner membrane potential. JC-1 is a lipophilic fluorescent probe that acts as the mitochondrial inner membrane potential (∆ψ_m_) indicator. In healthy cells with high ∆ψ_m_, JC-1 spontaneously forms J-aggregates, which have red fluorescence, while in unhealthy cells with low ∆ψ_m_, JC-1 remains in the monomeric form, which has a green fluorescence. We monitored the green monomeric JC-1 dye to track the increase in unhealthy cells ∆ψ_m_ following exposure to C26:0 at 25 µM. The JC-1 fluorescence imaging showed that the percentage of low ∆ψ_m_ cells increased with increasing concentrations of C26:0 (Figure 5A). We also quantitated the ratio of healthy (red) to unhealthy (green) cells using a fluorescent plate reader, which significantly decreased with higher concentrations of C26:0 (Figure 5B). The healthy/unhealthy fluorescence ratio was 11.8 ± 0.7 for vehicle control, 7.9 ± 0.5 for 25 µM C26:0 treated cells, and 6.5 ± 0.4 for 50 µM C26:0 treated cells. Our results further confirmed the mitochondrial toxic effects of VLCFA in oligodendrocytes. Treatment with 500 µM NAC reversed the C26:0-induced decrease in ∆ψ_m_. NAC was observed to effectively increase ∆ψ_m_, as evident by decreased JC-1 monomer fluorescence that showed the dead cells, increased red fluorescence indicating healthy cells (Figure 5A), and increased ratio of the healthy/unhealthy cells from 7.9 ± 0.5 (25 µM C26:0 treated group) to 9.5 ± 0.4 (25 µM C26:0 + 500 µM NAC co-treated group) (Figure 5B). These results indicate that NAC effectively targets mitochondria under conditions of VLCFA toxicity and improves mitochondrial function in 158 N cells.

### 3.5. VLCFA Increases Sensitivity to Addition of Oxidants in Oligodendrocytes

**Figure 6:**
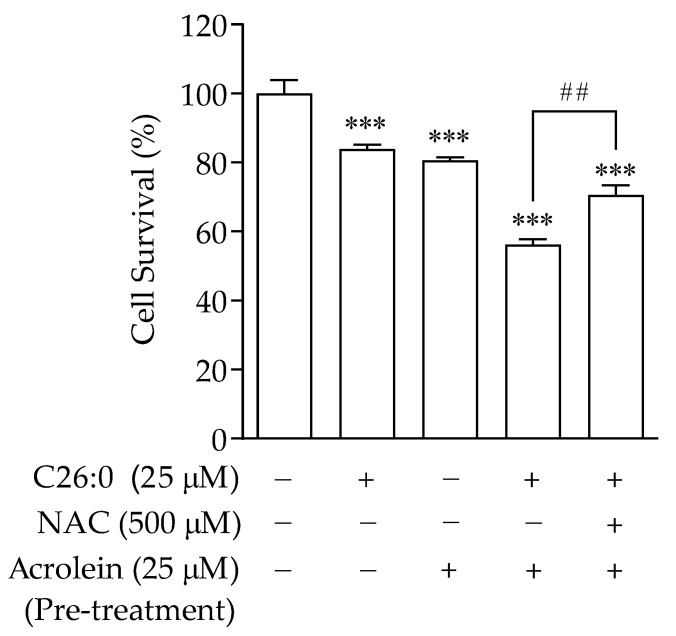
C26:0 increased sensitivity to additional oxidants such as acrolein in 158 N cells. Cell survival (in %) following treatments with C26:0 only (25 µM, 24 h), acrolein only (25 µM, 1 h), C26:0 (25 µM, 24 h) followed by acrolein (25 µM, 1 h), and C26:0 (25 µM)-NAC (500 µM) co-incubation for 24 h followed by acrolein (25 µM, 1 h) and control is shown. Cell survival was significantly reduced in all treated groups versus control (*** *p* < 0.001). Pre-treatment with C26:0 and subsequent incubation with acrolein at the same conditions profoundly decreased cell survival (56 ± 1.5%) compared to a single treatment of C26:0 or acrolein. Co-incubation of NAC with C26:0 before acrolein challenge increased the cell survival to 71 ± 3%, ## *p* < 0.01. Data were analyzed using ANOVA followed by Bonferroni’s test.

Although C26:0 at 25 µM concentration was observed to deplete mtGSH, increase superoxide anion levels, and impair mitochondrial inner membrane potential, there were no significant effects on intracellular ROS and total GSH levels, and this resulted only in moderate cell death. We hypothesized that the induced mitochondrial toxicity by pre-treatment of C26:0 could increase the sensitivity of these cells towards additional oxidant agents, such as those used during the preparatory regimen for stem cell transplantation. We used acrolein, the major reactive oxidant metabolite for the commonly used chemotherapeutic agent cyclophosphamide, to further induce oxidative stress in these cells. We measured cell viability following incubation with either threshold concentration of C26:0, or acrolein alone, or with acrolein following C26:0 pre-treatment. Oligodendrocytes incubated with low concentrations of C26:0 (25 µM, 24 h) or acrolein (25 µM, 1 h) showed modest cytotoxicity (84 ± 1% and 81 ± 1% cell survival, respectively). However, pre-treatment with C26:0 and subsequent incubation with acrolein under the same conditions profoundly increased cell toxicity (56 ± 1.5% cell survival), indicating a synergistic cytotoxic effect (Figure 6). These results confirmed our hypothesis that pre-treatment of C26:0 could increase sensitivity to oxidants such as acrolein. Next, we investigated whether NAC could protect C26:0 treated cells from such additional stress. Cells were co-incubated with NAC (500 µM) and C26:0 (25 µM) for 24 h before exposure with acrolein (25 µM) for 1 h. We observed that the addition of NAC significantly increased the cell survival to 71 ± 3% (*p* < 0.01). This could presumably be due to its effect on replenishing mtGSH and improving mitochondrial function, thereby desensitizing the cells to acrolein.

## 4. Discussion

**Figure 7:**
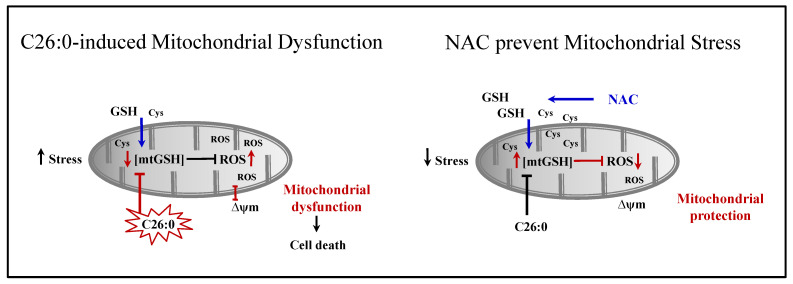
A hypothetical model depicting C26:0 injury in oligodendrocytes leading to the depletion of mtGSH. The loss of mtGSH predisposed cells to oxidant injury resulting in increased ROS accumulation, alteration of mitochondria inner membrane potential (∆ψ_m_), and finally cell death. NAC treatment prevented the mitochondrial toxicity of C26:0 by replenishing mtGSH and thus protected cells from additional oxidative stress and death.

In this study, we investigated the response of oligodendrocytes to VLCFA exposure and the potential benefit of using the antioxidant NAC. We demonstrate for the first time the depletion of mtGSH as an initial consequence of VLCFA treatment, which increases cellular sensitivity to additional oxidant stimuli. Further, we show that NAC can reverse the impaired mitochondrial function induced by VLCFA and offer protection in such cells from further oxidative damage (Figure 7). 

This study was designed to investigate how VLCFA accumulation leads to demyelination in ALD. The cerebral ALD phenotype is characterized by abnormal concentrations of VLCFAs, which accumulate in the white matter and cause rapid, progressive demyelination and subsequent neurodegeneration. Clinically, extensive demyelination in the white matter is associated with a poor prognosis in late-stage cerebral ALD [26,27]. Furthermore, increased concentrations of VLCFAs in the white matter correlate with the severity of ALD phenotypes [28]. Thus, the observed mitochondrial dysfunction, leading to the death of oligodendrocytes that synthesize myelin, is an important contributory factor to the inflammatory demyelination observed in cerebral ALD, and targeting this pathway is an attractive therapeutic option for such conditions. In vitro toxicity experiments in oligodendrocytes, incubated with VLCFA such as C26:0, offer molecular insights into the pathology of ALD. Previous studies have used a wide range of in vitro, in vivo, and clinical investigational systems, such as patient-derived fibroblasts [5,29,30], rat hippocampal cell cultures of oligodendrocytes and astrocytes [4], established oligodendrocyte cell lines [31,32], *Abcd1* genetic deficient mouse models [33], and patients showing the varied clinical presentations of ALD [34,35]. VLCFA was found to impact intracellular levels of reduced GSH and cell survival, increase reactive nitrogen and reactive oxygen species (ROS) production, impair lysosomal function, induce necrotic cell death, decrease ATP levels, and increase protein oxidative modifications in vitro [5,7,31,32].

Mitochondria, a vital organelle that is necessary for VLCFA degradation in concert with peroxisomes [36], has also been reported to be affected by the accumulation of VLCFA [6,7,37,38]. However, the exact mechanistic role of mitochondria in the cytotoxicity of VLCFA is still not fully understood. Mitochondrial abnormalities and impaired cross-talk between mitochondria and peroxisome were observed in human and mouse *Abcd1*-fibroblast cells [3]. Impaired mitochondrial oxidative phosphorylation and increased mtDNA oxidation were also observed in ALD patient-derived fibroblasts and *Abcd1*-mouse models [6]. Further, VLCFA affected the mitochondrial inner membrane potential, which was a likely underlying mechanism for “mitochondrial-based cell death” [4]. Similarly in our study, we observed decreased ATP production and mitochondrial inner membrane potential, as well as increased superoxide anion levels within mitochondria.

We investigated cellular response at threshold levels of VLCFA, which caused a minimal impact on cell survival. The concentration of VLCFA (C24:0 and C26:0) in patients with ALD is reported to be in the range of 1–5 µM in plasma [32] and 60 µM in the brain demyelinating plaques [5]. Hence, we exposed the cells to C26:0 at physiologically relevant concentrations of up to 25 µM for a short-term period. We hypothesized that low concentrations of VLCFA might predispose the cells to additional oxidative stress without causing significant changes in cell viability, becoming the first hit in the “three-hit hypothesis” [39]. Further, we theorized that the predisposition of cells towards oxidative stimuli by VLCFA is likely associated with mitochondrial dysfunction. This was supported by our observation that C26:0 by itself was not able to induce profound cell death, which occurred only after the second hit of oxidative stressors, which was the use of acrolein in our experiment. However, it should be noted that the marginal death (~15%) of oligodendrocytes that we observed following C26:0 exposure would have a significant impact on CNS function. Acrolein induces toxicity via oxidative stress and mitochondrial disruption [40]. Thus, the predisposition of cells to oxidative stress is probably closely related to the imbalanced redox system within the mitochondria. This is because the majority of ROS is produced within the mitochondrial respiratory chain as byproducts of ATP generation [41], and the imbalance between ROS and antioxidants within the mitochondria might be one of the crucial pathways leading to its dysfunction, further triggering the oxidative stress cascade. A recent study reported that treating 158 N cells with C26:0 at concentrations of up to 20 µM for 24 and 48 h induced cell death due to mitochondrial dysfunction and oxidative stress [42].

Among the arsenal of protective antioxidants, mitochondrial GSH (mtGSH) has emerged as the mainline of defense against oxidative stress by maintaining an optimal redox environment within the organelle [43]. Loss of mtGSH predisposes the cell towards oxidant-induced injuries including cerebral ischemia [44] and hypoxia [45]. Interestingly, we observed depletion in mtGSH without a significant decrease in total cellular GSH at threshold VLCFA concentrations, suggesting mtGSH to be the first target for VLCFA toxicity. Although mtGSH composes a minor fraction of total cellular GSH (10–15%) [46], it is observed to be more sensitive to VLCFA treatment compared to total cellular GSH.

Strategies aimed at the restoration of mtGSH could desensitize the cells to VLCFA, protect them from further oxidant injury, and in turn improve cell survival. NAC, a well-known antioxidant, provides the rate-limiting substrate, cysteine for GSH synthesis, and protects cells from free radical damage. NAC is widely used to combat oxidative stress and mitochondrial dysfunction [47,48,49]. For instance, NAC has been shown to effectively reverse the 3-nitropropionic acid-induced inhibition of mitochondrial complexes II, IV, and V in a Huntington’s disease rat model [47] and to attenuate *N*-methylprotoporphyrin-induced inhibition of cytochrome oxidase assembly in Alzheimer’s disease fibroblasts [48]. In this study, we found that NAC could act at the mitochondrial level, replenishing mtGSH, reducing superoxide levels within mitochondria, and increasing the inner membrane potential, thereby relieving the first hit of oxidative stress in the ALD disease oligodendrocyte model. Further assessments are needed to confirm our findings in ALD patient-derived fibroblasts, animal models and astrocyte clones derived from these models [50]. Additionally, we show that VLCFA-treated oligodendrocytes had lower antioxidant defenses and hence, increased sensitivity to chemical insults resulting in irreversible cell death. NAC treatment reinstated the redox balance in mitochondria, improved its function, and thus protected these cells from further oxidant injury. Results from our studies support NAC as a potential therapy for ALD. In addition, our study supports in vitro, in vivo, and clinical approaches wherein NAC is combined with mitochondrial cofactors (vitamin C, vitamin E, and coenzyme Q) to enhance its therapeutic benefits [51]. Future studies should aim at further characterizing the precise mechanisms of NAC in restoring mitochondrial function and use various combinations of antioxidants to further optimize the protective effects on mitochondria.
